# Induced seismicity

**DOI:** 10.1038/s41598-024-79796-z

**Published:** 2024-11-23

**Authors:** Gillian R. Foulger, Longjun Dong

**Affiliations:** 1 Durham University, Durham, UK; 2 Ocean University of China, Qingdao, China; 3 Central South University, Changsha, China

**Keywords:** Environmental impact, Natural hazards

## Abstract

Human-induced earthquakes are an issue of foremost significance affecting multiple industries such as geothermal, oil and gas, water, mining, and waste disposal. Current research addresses global monitoring of induced earthquakes, case histories, improving monitoring techniques, understanding the physical mechanisms involved, forecasting, mitigating risk, predicting the magnitude of the largest possible earthquake, and assessing impacts and stakeholder opinion. This Guest Editor Collection presents over 20 research papers summarizing the current state of knowledge throughout all aspects of the subject, and comprises an excellent resource for stakeholders.

Human-induced earthquakes date back as far as people have engineered the Earth’s crust, *e.g.*, by mining^1^. See also https://inducedearthquakes.org/. Traditionally, they have been viewed as an unavoidable industrial hazard – an inevitable cost of progress. More recently, however, public tolerance for induced earthquakes has reduced. Infrastructure is built on a larger scale, it may be installed in dense urban areas with no prior history of earthquakes and existing buildings may not meet earthquake-resilience standards.

Significant challenges are understanding the mechanisms of induced earthquakes and developing management methods^1^. Induced earthquakes must be managed or projects may be prevented from going ahead, hampering economic development and the transition to “green” energy. Earthquakes are fundamentally unpredictable, fractal in size-distribution, and chaotic in occurrence. Prevention and prediction thus presents Earth scientists and engineers with challenges.

The Induced Seismicity Collection of *Scientific Reports*contains over 20 papers that provide an update of all aspects of this critical subject. Contributions include analyses of particular case histories e.g., the High Agri Valley, Italy^2^, using induced earthquakes for subsurface exploration e.g., at The Geysers geothermal field, California^3^, earthquake forecasting e.g., in Oklahoma^4^, earthquake mitigation^5^, induction processes^6^, and the application of small-scale laboratory^7^ and large-scale space-based^8^ techniques to the subject.

Fluid extraction for electricity generation at The Geysers geothermal field, California, has induced prolific earthquake seismicity for over 50 years. This provides a reliable source of earthquake data that enables highly designed datasets to be gathered. Staszek et al.^3^ built on this to develop ways to precisely delineate subsurface structures likely to comprise underground fluid pathways. Zhang et al.^9^ analyzed earthquakes in the Hutubi underground gas storage facility and demonstrated that strain localization due to non-uniform gas injection was responsible for the earthquakes. Both these studies describe forefront approaches to hazard mitigation strategies.

Earthquake forecasting is the main objective of three papers in the Collection^4,9,10^. Qin et al.^4^ used machine learning to forecast induced seismicity associated with wastewater injections in Oklahoma. Li et al.^10^ addressed the problem of forecasting the largest earthquake that may be induced–a critical question since this may be the most damaging one. Zhang et al.^11^ analyze the variation in time of *b*-value, D-value, and the spatial correlation length for earthquakes near the Three Gorges Reservoir. They found that variations in time of the *b*-value could help predict earthquakes with magnitudes ~ M4.0. These papers highlight that multiple different approaches may be required for earthquake forecasting.

Improvements in detecting seismic events is the subject of four papers. Herath et al.^12^develop a single-station earthquake location method based on travel-time back-projection, Rodriguez and Myklebust^13^ present a learning method for seismological parameters to detect and categorize sources rapidly, and Stabile et al.^2^ demonstrate a single-station template matching algorithm.

Risk control is critical for minimizing hazard from induced earthquakes and several papers focus on this issue. Muntendam-Bos and Grobbe^14^ analyze the size distribution of earthquakes and study their spatial and temporal variability at Groningen, Netherlands. Schultz et al.^15^ investigate the applicability to induced seismicity of Båth’s law, which models the decay in earthquake numbers in sequences, to understand how induced earthquake sequences end. Yaghoubi et al.^16^ demonstrate a probabilistic method to assess fault slip trends and Eyre et al.^8^ analyze the role of slow-slip events in fault activation from ground deformation observations. The seismic hazard associated with fluid injection is the particular focus of papers by Bondarenko et al.^17^, who introduce a coupled hydro-mechanical numerical approach, and Rodríguez-Pradilla et al.^18^ who show that, for wells in the Western Canada Sedimentary Basin, there is a limit to the amount of tectonic strain energy that can contribute to a runaway rupture.

Contributions to understanding the earthquake-genesis process include papers by Oye et al.^7^, who describe laboratory induced-seismicity experiments and Kivi et al.^19^ who apply a coupled thermal/water/mechanical model to a hot sedimentary aquifer. Chang et al.^20^ study fluid migration at depth along basement faults at wastewater injection sites, and conclude that slow diffusion along faults can limit seismicity and seismic potential may be quickly reduced by reducing injection volumes.

Rapid urbanization can significantly impact subsurface stress and seismic hazard. Research at the interface of science and sociology is thus ascendant. Papers in the Collection representing this theme include that by Tiwari et al.^21^ who show that modulation of the seismic rate and the stability of basement thrust faults around Delhi, India are linked to both urbanization and decadal changes in groundwater. Evensen et al.^22^ explore public attitudes in the UK to the moratorium on shale gas development there and found that there was little support for changing this policy. Studies of this kind are clearly of key importance to potentially seismogenic industries and should arguably be routine moving forward.

This Collection represents a valuable milestone summary of human-induced earthquake research. It provides an excellent starting point for new scholars entering the field and individuals needing a current update. It offers an excellent resource for the broad range of stakeholders including researchers, industrialists, environmentalists and local residents. The Collection also illustrates that, despite the considerable progress made in recent years, many challenges still remain. The most important of these include understanding why induced seismicity is sometimes delayed and distant from the causative activity, developing methods for distinguishing natural and induced earthquakes, and estimating the likely maximum magnitude for earthquakes in a given project. Improvements in risk estimation to infrastructure from induced earthquakes are also urgently needed, along with improved industry standards for managing induced earthquakes. Hopefully this Collection will encourage more researchers and others to pick up these challenges and engage in this pivotal subject.
